# Memory stem T cells modified with a redesigned CD30‐chimeric antigen receptor show an enhanced antitumor effect in Hodgkin lymphoma

**DOI:** 10.1002/cti2.1429

**Published:** 2022-11-03

Carmen Alvarez‐Fernández, Laura Escribá‐Garcia, Ana Carolina Caballero, Eva Escudero‐López, Cristina Ujaldón‐Miró, Rosanna Montserrat‐Torres, Paula Pujol‐Fernández, Jorge Sierra & Javier Briones

*Clinical & Translational Immunology* 2022; **11**: e1429.

**Correction to**: Clin Trans Immunol 2021; **10**: e1268. https://doi.org.10.1002/cti2.1268. Published online 29 April 2021.

Ana Carolina Caballero's affiliation has been corrected. The complete affiliation is as follows:

^4^Department of Medicine, Autonomous University of Barcelona, Barcelona, Spain.

The authors apologise for this error.

